# Potential Transdiagnostic Lipid Mediators of Inflammatory Activity in Individuals With Serious Mental Illness

**DOI:** 10.3389/fpsyt.2021.778325

**Published:** 2021-11-26

**Authors:** Ulrika Hylén, Aidan McGlinchey, Matej Orešič, Susanne Bejerot, Mats B. Humble, Eva Särndahl, Tuulia Hyötyläinen, Daniel Eklund

**Affiliations:** ^1^University Health Care Research Center, Faculty of Medicine and Health, Örebro University, Örebro, Sweden; ^2^School of Medical Sciences, Faculty of Medicine and Health, Örebro University, Örebro, Sweden; ^3^Inflammatory Response and Infection Susceptibility Centre, Örebro University, Örebro, Sweden; ^4^Man-Technology-Environment Research Centre, School of Science and Technology, Örebro University, Örebro, Sweden

**Keywords:** mental disorder (disease), schizophrenia, autism spectrum disorder (ASD), obsessive-compulsive disorder, lipidomics, inflammation

## Abstract

Mental disorders are heterogeneous and psychiatric comorbidities are common. Previous studies have suggested a link between inflammation and mental disorders. This link can manifest as increased levels of proinflammatory mediators in circulation and as signs of neuroinflammation. Furthermore, there is strong evidence that individuals suffering from psychiatric disorders have increased risk of developing metabolic comorbidities. Our group has previously shown that, in a cohort of low-functioning individuals with serious mental disorders, there is increased expression of genes associated with the NLRP3 inflammasome, a known sensor of metabolic perturbations, as well as increased levels of IL-1-family cytokines. In the current study, we set out to explore the interplay between disease-specific changes in lipid metabolism and known markers of inflammation. To this end, we performed mass spectrometry-based lipidomic analysis of plasma samples from low-functioning individuals with serious mental disorders (*n* = 39) and matched healthy controls (*n* = 39). By identifying non-spurious immune-lipid associations, we derived a partial correlation network of inflammatory markers and molecular lipids. We identified levels of lipids as being altered between individuals with serious mental disorders and controls, showing associations between lipids and inflammatory mediators, e.g., osteopontin and IL-1 receptor antagonist. These results indicate that, in low-functioning individuals with serious mental disorders, changes in specific lipids associate with immune mediators that are known to affect neuroinflammatory diseases.

## Introduction

Mental disorders are common and constitute a major, global burden of disease as well as a challenge to healthcare, with a number of unmet needs (1). People with mental disorders have two-to three-fold higher risk of premature death compared to the general population (2). Since aetiological factors and pathophysiological mechanisms are largely unknown, mental disorders are still classified and diagnosed based on signs and symptoms (3), and not on biomarkers. Symptomatology often overlaps between various mental disorders, adding to a high degree of comorbidity, especially in more severe cases (4). A transdiagnostic approach has therefore gained increased support in the field of psychiatry (5), especially in studies of chronic and seriously ill individuals. This approach aims to delineate underlying pathophysiological processes across traditional diagnostic groups (6).

Inflammation has previously been associated with mental disorders, such as depression, bipolar disorder, schizophrenia spectrum disorder (SSD), autism spectrum disorder (ASD), and obsessive-compulsive disorder (OCD) (7–10). Patients suffering from these mental disorders tend to have increased levels of circulating cytokines and increased microglial activity in the central nervous system (CNS), suggesting that inflammation may contribute to the onset, or the chronicity, of mental disorders (11–14).

Depression, bipolar disorder, SSD and ASD have also been associated with alterations in the lipidome (15, 16), especially of phospholipids, such as lysophosphatidylcholines (LPCs), phosphatidylcholines (PCs), phosphatidylethanolamines (PEs), and sphingomyelins (SMs) (17–19). Such alterations in the lipidome have also been linked to inflammatory processes in other diseases, such as type 2 diabetes mellitus and cardiovascular diseases (20). However, few studies have focused on this link in mental disorders (15).

Inflammasome activation, Toll-like receptor (TLR) signalling and immune cell differentiation are also implicated at the juncture of inflammation and metabolism (21). For example, oxidative stress and mitochondrial reactive oxygen species (ROS) are well-known triggers for NLRP3 activation (22). In addition, saturated fatty acids and oxidised phospholipids may induce Nod-like receptor 3 (NLRP3) activation and the production of interleukin (IL)-1 family cytokines through ROS-dependent mechanisms (23–25). In a recent study by our group, increased activity of genes associated with the NLRP3 inflammasome (*CASP1, NLRP3, PYCARD, IL1B, IL1RN*), as well as increased levels of IL-1-family cytokines (IL-1 receptor antagonist (IL1Ra), IL-18), were found in a transdiagnostic cohort with low-functional individuals with mental disorders (26). In the current study, we aim to investigate the link between inflammatory pathways, including the NLRP3 inflammasome, selected markers of inflammation, previously identified markers of neuroinflammatory events (27), and the lipidome in low-functioning individuals with serious mental disorders.

## Materials and Methods

### Participants

In this cross-sectional study, individuals with serious mental disorders were recruited from psychiatric clinics in Örebro County and the surrounding regions (Stockholm, Karlstad and Lidköping) in Sweden. The inclusion criteria for the patient group were: age 16–50 years and being diagnosed with at least one of four serious mental illnesses: SSD, ASD, OCD, or non-suicidal self-injury disorder (NSSID), allowing further comorbidity. These primary disorders were selected due to their course with chronicity or frequent relapses, and their association with inflammation in previous research. The Clinical Global Impression Severity scale (CGI-S) was used, since it assesses clinical severity irrespective of diagnosis, and only participants scoring at least markedly ill, i.e., with a CGI-S score of 5 or more, were included in the study. Exclusion criteria were any neurological autoimmune disorders and/or an ongoing infection at the time of blood sampling. All individuals with serious mental disorders were recruited between November 2016 and June 2018.

Healthy controls were recruited in Örebro, consecutively after patient inclusion to match age and gender (Table 1). Exclusion criteria for controls were any psychiatric or medical disorder, yet three had been diagnosed with physical conditions (psoriasis, asthma and allergy to nuts, respectively), i.e., inflammatory-related disorders which theoretically could affect our results. However, all three were in clinical remission at the time of the study and none received medical treatment.

**Table 1:** Demographics of participants.

	**Patients**	**Controls**
	***n*** **= 39**	***n*** **= 39**
**General parameters**		
Sex (M/F)	15/24	15/24
Age, mean (range)	28 (16–47)	28 (16–45)
BMI, mean (SD)	26.2 (5.9)	23.2 (2.7)
Educational level <12 years	13	4
Educational level ≥12 years^*^	19	34
Working	3	26
Student	6	13
Permanent sick leave	30	0
**Medical diagnoses**		
Asthma	2	1
Allergy	1	1
Diabetes type II	1	-
Coeliac disease	1	-
Hypothyroidism	2	-
Gastric bypass surgery	2	-
Epilepsy	1	-
Psoriasis	-	1
Brain malformation	1	-

* *Missing values, 7 patients, 1 control*.

### Diagnostic Assessment Prior to Inclusion

All participants with a mental disorder had been diagnosed according to the Diagnostic and Statistical Manual of Mental Disorders (DSM-IV) by a board-certified psychiatrist prior to enrolment. In addition, all psychiatric and medical records were available to the researchers throughout the study. Notably, assessment for ASD includes a comprehensive interview with parents of the individual on early signs of autism, in addition to extensive interviews and cognitive tests performed by experienced psychiatrists and psychologists, according to Swedish diagnostic routines.

### Procedures and Diagnostic Assessments Within the Study

For diagnostic validation, a board-certified psychiatrist interviewed each potential participant for 3 to 4 h at one single appointment, at Örebro University hospital or at the home of the participant. Psychiatric comorbidities were assessed with the Mini International Neuropsychiatric Interview (M.I.N.I. version 7) (28) and the Clinician Administered Non Suicidal Self-Injury Disorder Index (CANDI) (29). Global severity was assessed with the Clinical Global Impression Severity scale (CGI-S), and the Global Assessment of Functioning (GAF). CGI-S is a single-value measurement of severity (1 = not at all ill, 2 = borderline mentally ill, 3 = mildly ill, 4 = moderately ill, 5 = markedly ill, 6 = severely ill and 7 = extremely ill) (30). The GAF metric measures overall psychiatric dysfunction with separate ratings for symptoms and disability (1 = extremely impaired and 100 = extremely high functioning) (31). The patients also rated their own severity of illness on the self-rating version of CGI-S, Patient Global Evaluation (PGE). An additional assessment of disability was performed with the self-administered, 12-item version of the WHO Disability Assessment Schedule (WHODAS 2.0) in order to include the patient's own view on the extent of their disability. The complex method of scoring was used, for which an algorithm is utilised to calculate a total score (0 = no disability and 100 = full disability), with the resulting score indicating the percent of disability (32).

Depression was assessed using the nine item Patient Health Questionnaire (PHQ-9) (33), and personality disorder traits were measured with The Personality Inventory for DSM-5, brief form (PID-5-BF) (34); PHQ-9 and PID-5-BF are both self-rated. To confirm the severity of an ASD diagnosis, we used the Clinician-Rated Severity of Autism Spectrum and Social Communication Disorders and, to confirm the severity of SSD, the Clinician-Rated Dimensions of Psychosis Symptom Severity was used; both are DSM-5 derived measures (35). Further, the severity of the OCD diagnosis was determined with the Global Obsessive-Compulsive Scale (NIMH-GOCS) (36), and the Deliberate Self-Harm Inventory (DSHI-9) was used for assessment of NSSID (37). After the interview, each participant was assigned a primary diagnostic group: SSD, ASD, OCD, or NSSID in addition to the comorbidities.

The controls were also interviewed with the above-described work-up by a registered nurse, trained in the assessment to exclude individuals with current or previous psychiatric disorder.

### Sample Collection and Preparation

At the same day and location as the psychiatric interview, peripheral whole blood was collected from participants between 8 and 10 a.m., following an overnight fast. A total of 86 mL was drawn in eight, 10 mL EDTA tubes, one 4 mL EDTA tube, and one PAXgene^®^ Blood RNA tube. The 4 mL EDTA tubes were placed on ice directly after sampling. The tubes were centrifuged for 15 min at 1500 x g and plasma were then separated from the whole blood, distributed into aliquots of 2 ml and stored at−80 °C until further analysis.

### Measuring Inflammatory Markers

Methods used for quantifying gene expression levels of inflammatory genes and circulating levels of the presented cytokines have already been published (26). Briefly, gene expression analysis was performed by extracting RNA from whole blood using the PAXgene^®^ system, and subsequent qPCR was performed using the TaqMan Fast Universal PCR Master Mix (Applied Biosystems) and TaqMan Gene Expression Assays (Applied Biosystems). The relative quantity was determined for each target gene using a serial four-fold dilution standard curve from stimulated peripheral blood mononuclear cells (PBMCs). All samples were run in duplicate with an accepted coefficient of variation of < 15%. Samples with threshold cycle (Ct) > 35 were not used for quantification. All target genes were normalised using the geometric mean of the reference genes *HPRT1* and *TBP*. For quantification of circulating cytokines and chemokines in plasma, reagents and instrumentation for electrochemiluminescence (ECL) multiplex assay (U-Plex Biomarker 1-Human) from Meso Scale Diagnostics (MSD), Rockville, MD, was used, except for circulating IL-1β, where the SIMOA (Single Molecule Array) digital immunoassay platform was used (Quanterix, Lexington, MA, USA).

### Lipidomics Analysis

The serum lipids were extracted using a modified version of the previously published Folch procedure (38). Shortly, 10 μL of 0.9 % NaCl and 120 μL of CHCl3: MeOH (2:1, v/v), containing 2.5 μg mL-1 internal standards solution (for quality control and normalisation purposes), were added to 10 μL of each plasma sample. The standard solution contained the following compounds: 1,2-diheptadecanoyl-sn-glycero-3-phosphoethanolamine [PE(17:0/17:0)], N-heptadecanoyl-D-erythro-sphingosylphosphorylcholine [SM(d18:1/17:0)], N-heptadecanoyl-D-erythro-sphingosine [Cer(d18:1/17:0)], 1,2-diheptadecanoyl-sn-glycero-3-phosphocholine [PC(17:0/17:0)], 1-heptadecanoyl-2-hydroxy-sn-glycero-3-phosphocholine [LPC(17:0)] and 1-palmitoyl-d31-2-oleoyl-sn-glycero-3-phosphocholine [PC(16:0/d31/18:1)], were purchased from Avanti Polar Lipids, Inc. (Alabaster, AL, USA), tripalmitin- Triheptadecanoylglycerol [TG(17:0/17:0/17:0)] (Larodan AB, Solna, Sweden). The samples were vortex-mixed and incubated on ice for 30 min after which they were centrifuged (9,400 × g, 3 min, 4° C). 60 μL from the lower layer of each sample was then transferred to a glass vial with an insert and 60 μL of CHCl3: MeOH (2:1, v/v) was added to each sample. The samples were then stored at−80°C until analysis.

Calibration curves using 1-hexadecyl-2-(9Z-octadecenoyl)-sn-glycero-3-phosphocholine {PC[16:0/18:1(9Z)]}, 1-(1Z-octadecenyl)-2-(9Z-octadecenoyl)-sn-glycero-3-phosphocholine [PC(16:0/16:0)], 1-octadecanoyl-sn-glycero-3-phosphocholine [LPC(18:0)], (LPC18:1), PE (16:0/18:1), (2-aminoethoxy){(2R)-3-hydroxy-2-[(11Z)-octadec-11-enoyloxy]propoxy} phosphinic acid [LysoPE(18:1)], N-(9Z-octadecenoyl)-sphinganine {Cer [d18:0/18:1(9Z)]}, 1-hexadecyl-2-(9Z-octadecenoyl)-sn-glycero-3-phosphoethanolamine [PE (16:0/18:1)] from Avanti Polar Lipids, Inc., 1-Palmitoyl-2-Hydroxy-sn-Glycero-3-Phosphatidylcholine [LPC(16:0)] and 1,2,3 trihexadecanoalglycerol (TG16:0/16:0/16:0), 1,2,3-trioctadecanoylglycerol [TG(18:0/18:0/18:0)] and ChoE(18:0), 3β-Hydroxy-5-cholestene 3-linoleate [ChoE(18:2)] from Larodan, were prepared to the following concentration levels: 100, 500, 1,000, 1,500, 2,000 and 2,500 ng mL^−1^ (in CHCl3:MeOH, 2:1, v/v) including 1,000 ng mL^−1^ of each internal standard.

The samples were analysed using ultra-high-performance liquid chromatography quadrupole time-of-flight mass spectrometry method (UHPLC-QTOFMS), which has been presented in detail previously (39). Briefly, the UHPLC system used in this study was a 1,290 Infinity system from Agilent Technologies (Santa Clara, CA, USA). The system was equipped with a multi-sampler (maintained at 10°C), a quaternary solvent manager and a column thermostat (maintained at 50°C). Separations were performed on an ACQUITY UPLC® BEH C18 column (2.1 × 100 mm, particle size 1.7 μm) by Waters (Milford, USA).

The mass spectrometer coupled to the UHPLC was a 6,545 QTOF instrument from Agilent Technologies interfaced with a dual jet stream electrospray (dual ESI) ion source. All analyses were performed in positive ion mode and MassHunter B.06.01 (Agilent Technologies) was used for all data acquisition. mass spectrometry data processing was performed using open-source software MZmine (v.2.1834). The following steps were applied in the processing: (i) Crop filtering with a m/z range of 350–1200 m/z and retention time (RT) range of 2.0 to 15.0 min, (ii) Mass detection with a noise level of 1,000, (iii) Chromatogram builder with a min time span of 0.08 min, min height of 1,200 and a m/z tolerance of 0.006 m/z or 10.0 ppm, (iv) Chromatogram deconvolution using the local minimum search algorithm with a 70 % chromatographic threshold, 0.05 min minimum RT range, 5 % minimum relative height, 1,200 minimum absolute height, a minimum ration of peak top/edge of 1.2 and a peak duration range of 0.08-5.0, (v) Isotopic peak grouper with a m/z tolerance of 5.0 ppm, RT tolerance of 0.05 min, maximum charge of 2 and with the most intense isotope set as the representative isotope, (vi) Peak list row philtre keeping only peaks with a minimum of 10 peaks in a row, (vii) Join aligner with a m/z tolerance of 0.009 or 10.0 ppm and a weight for of 2, a RT tolerance of 0.1 min and a weight of 1 and with no requirement of charge state or ID and no comparison of isotope pattern, (viii) Peak list row philtre with a minimum of 53 peaks in a row (=10% of the samples), (ix) Gap filling using the same RT and m/z range gap filler algorithm (with an m/z tolerance of 0.009 m/z or 11.0 ppm, 10). Identification of lipids using a custom database search (with an m/z tolerance of 0.009 m/z or 10.0 ppm and a RT tolerance of 0.1 min, 15). Normalisation using internal standards [PE (17:0/17:0), SM (d18:1/17:0), Cer (d18:1/17:0), LPC (17:0), TG (17:0/17:0/17:0) and PC (16:0/d30/18:1)] for identified lipids and closest ISTD for the unknown lipids, followed by calculation of the concentrations based on lipid-class concentration curves.

Quality control was performed throughout the dataset by including blanks, pure standard samples, extracted standard samples and control plasma samples. Relative standard deviations (%RSDs) for lipids in the pooled quality control (*n* = 5) were on average 20.0 %.

### Statistical Analysis

All statistical analyses were carried out using the statistical programming language, R (40). The pre-processing of the dataset, for both lipidomics data and for the measurements of the inflammatory factors, involved: (1) missing values or values less than zero in the data were replaced with zeroes, (2) all zeroes were then replaced with imputed half-minimums (for each variable, the minimum value was found, and half of this value was used), (3) all values were log2 transformed and (4) values for each variable was scaled to zero mean and unit variance (autoscaling). In order to ascertain the changes in lipid and inflammatory factor levels due to the presence of mental disorder, the fold-change of all lipids and inflammatory factors was calculated as a simple subtraction of the log 2, autoscaled values for all participants with mental disorder minus their respective, matched controls. This is referred to hereafter as the “fold change matrix” and was used for all subsequent analyses.

For correlation analysis of all lipids and inflammatory factors, Spearman correlations were calculated pairwise on the fold change matrix. For visualisation, the *corrplot* R package (version 0.84) was used (41).

**Figure 1:**
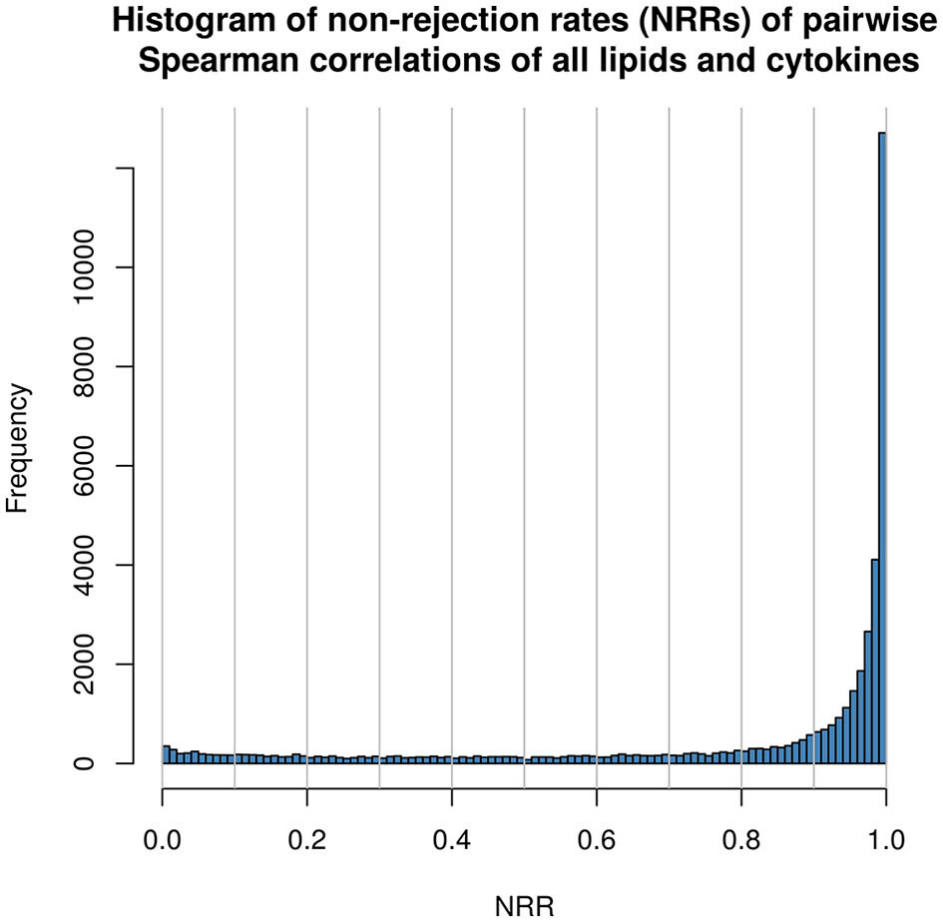
Histogram displaying frequency of non-rejection rates (NRRs) as calculated pairwise between all lipids and inflammatory mediator in the study.

For network analysis and projection, the *qpNrr* function from the *qpgraph* R package (version 2.16.0) (42) was run with default parameters to estimate the non-rejection rates (NRRs) of all pairwise correlations in the aforementioned matrix, to identify likely-spurious associations. A histogram depicting the frequencies of NRRs was generated from this matrix of NRRs in order to judge a suitable threshold to conservatively retain associations of interest and eliminate spurious ones (Figure 1). Thereby, the fold change matrix was filtered by removing from it all relationships which, in the NRR matrix, did not pass the set threshold value (NRR ≤ 0.1), and the remaining associations were used to generate the edges for network projection.

To provide the desired inflammatory mediator-centric approach and avoid cluttering the network figure to the point of illegibility with lipid-lipid interactions, the aforementioned table of non-spurious associations was subsequently trimmed to contain only associations between inflammatory mediators and other elements (those being other inflammatory mediators or lipids). This essentially removed, for clarity, lipid-lipid interactions which did not involve any inflammatory mediator.

**Figure 2:**
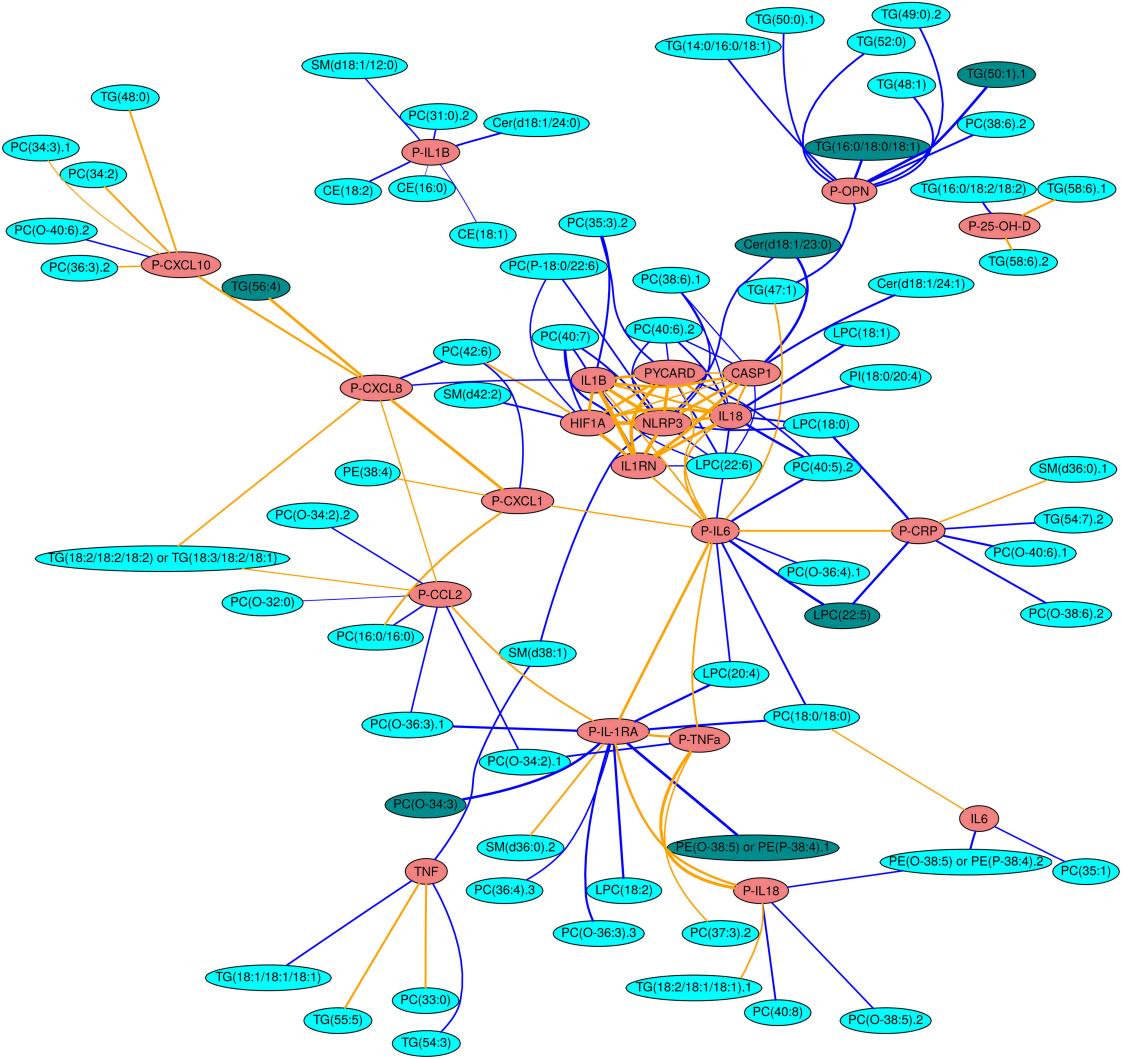
Network projection of all non-spurious (NRR < 0.1) correlations between immune mediators and lipids. Red nodes indicate gene expression data or plasma protein data (P-) of immune mediators and cyan nodes indicate circulating lipids. The darker shade of cyan indicates the lipids with the strongest (≥0.5), correlations across all patients (see Methods). Blue lines denote negative correlations, whilst orange lines denote positive correlations. The thickness of the lines is scaled to the magnitude of the correlation.

For this network projection, the *Rgraphviz* R package (version 2.26.0) (43) was used to generate network topography plots. Node and edge properties for these network graphs were generated in a custom fashion (see the legend of Figure 2). The positioning of nodes in the network figure is generated by the *Rgraphviz* package itself, and the clearest layout was obtained using the “fdp” layout option. Manual reduction of the size of the figure was carried out where certain peripheral nodes were drawn with anomalously long edges as an artefact of automated network projection.

Following the observation of fold changes in lipids and inflammatory mediators vs. matched controls (see **Figure 4**), it became apparent that there was noteworthy heterogeneity in the changes in lipid / inflammatory mediators levels vs. matched controls. Therefore, analysis was also carried out to ascertain if any lipids / inflammatory mediators were consistently raised or lowered in all participants with mental disorder vs. their matched controls. These consistently-changing lipids were identified as the strongest associations (Spearman correlation ≥ 0.5 or ≤ −0.5) in the NRR-filtered, inflammatory mediators-centric matrix of NRR correlations. These could then be said to be strong, non-spurious, disease-associated associations which were the most consistent across all participants with mental disorder. These strongest candidate lipid biomarkers of disease were then plotted as bean plots, using the *beanplot* package (44) for R, depicting the change in their level between controls and patients (**Figure 5**).

Consideration was given to any potential effects of antipsychotic medication on the measured features, thus significance testing and multiple hypothesis correction were performed by either Benjamini-Hochberg or Bonferroni correction. To this end, generalised linear models (GLM) were built to examine if there were any relationships between the measured lipids / inflammatory factors and a flag indicating the use of antipsychotic medication.

### Ethics and Consent

All participants received verbal and written information about the study, adapted to the low functioning of the participants, and signed an informed consent prior to being enrolled in the study. The study was approved by the Regional Ethical Review Board in Uppsala, Sweden (2016/091).

## Results

### Cohort Demographics and Clinical Characteristics

This study drew upon a cohort of 39 low-functioning individuals with severe mental disorders (ages 16–47 years) and 39 age and sex matched controls (Table 1).

The psychiatric comorbidity in the patient cohort was substantial; 64 % fulfilled the criteria for major depression (3). Furthermore, the majority (59 %) met the diagnostic criteria of a least five different psychiatric diagnoses, eight participants fulfilled six or more diagnoses, and one participant met the criteria for 13 diagnoses. Of these diagnoses, four participants fulfilled a diagnosis for alcohol use disorder and one for substance-related and addictive disorder (cannabis). The mean age of onset for the whole group was 12 years old (range 4–33), and 22 patients had a childhood onset (before 13 years). A summary of included participants is shown in Table 2.

**Table 2:** Clinical characteristics of patients.

	**All patients**	**SSD**	**ASD**	**OCD**	**NSSID**
	***n*** **= 39**	***n*** **= 14**	***n*** **= 8**	***n*** **= 9**	***n*** **= 8**
**Diagnostic assessment**					
Number of psychiatric diagnoses (median, range)	5 (1–13)	6 (2–10)	3 (1–10)	5 (1–11)	6 (3–13)
Current depression, n (%)	25 (64)	7 (50)	4 (50)	6 (67)	8 (100)
PHQ-9 (median, range)	18 (0–26)	21 (5–26)	16 (4–23)	14 (4–20)	21 (0–25)
PID-5-BF (median, range)	27 (7–51)	32 (16–49)	26 (7–30)	23 (9–51)	32 (16–43)
CRD-PSS (median, range)	5 (0–25)	9 (3–25)	4 (0–14)	3 (0–6)	5 (0–15)
NIMH-GOCS (median, range)	2 (0–13)	0 (0–10)	1 (0–5)	11 (3–13)	1 (0–7)
DSHI-9 (median, range)	6 (0–48)	1 (0–27)	0 (0–13)	1 (0–12)	30 (22–48)
CANDI (yes/no)	11/22^a^	3/9^b^	1/5^b^	1/7^c^	6/1^c^
WHODAS 2.0 (median, range)	69 (27–100)	65 (41–100)	83 (27–100)	73 (58–85)	64 (33–81)
CGI-S (median, range)	6 (3–7)	5 (3–7)	5 (4–6)	6 (5–7)	6 (5–7)
PGE (median, range)	5 (2–7)	5 (3–7)	6 (3–7)	5 (4–6)	5 (2–6)
GAF (median, range)	42 (11–61)	40 (11–61)	46 (22–60)	45 (35–57)	42 (25–51)
**Medications** **(***n***)**					
SRI	16	3	3	6	4
MAO inhibitors	3	0	0	0	3
Central stimulants	3	0	1	1	1
Antipsychotics (other than clozapine)	17	9	4	1	3
Clozapine	4	4	0	0	0
Lithium	4	2	0	1	1
Anticonvulsants	10	5	1	1	3
GABA-ergic sedatives	11	5	2	2	2
Histamine blockers	12	4	3	1	4
Anticholinergics	5	3	2	0	0
**Course of the disease**					
First episode of the disorder	2	2	0	0	0
Relapse and treated in inpatient unit	19	9	2	3	5
Relapse, but not in inpatient care	9	1	2	2	4
Chronic course	9	2	4	2	1

*PHQ-9, Patient Health Questionnaire (PHQ) (range 0–27), Scores of 5, 10, 15, and 20 represent cut-points for mild, moderate, moderately severe, and severe depression). PID-5-BF, The Personality Inventory for DSM-5 Short Form. CRD-PSS, Clinician rated dimensions of Psychosis Symptoms Severity. NIMH-GOCS, Global Obsessive-Compulsive Scale. DSHI-9, Deliberate Self-Harm Inventory−9 item version; maximum score 54. CANDI, Clinician Administered Non-Suicidal Self-Injury Disorder Index. CGI-S, Clinical Global Impression Scale–Severity. PGE, Patient Global Evaluation. GAF, Global Assessment of Functioning*.

a *5 missing*,

b *2 missing*,

c *1 missing*.

### Inflammation-Lipid Interactions

**Figure 3:**
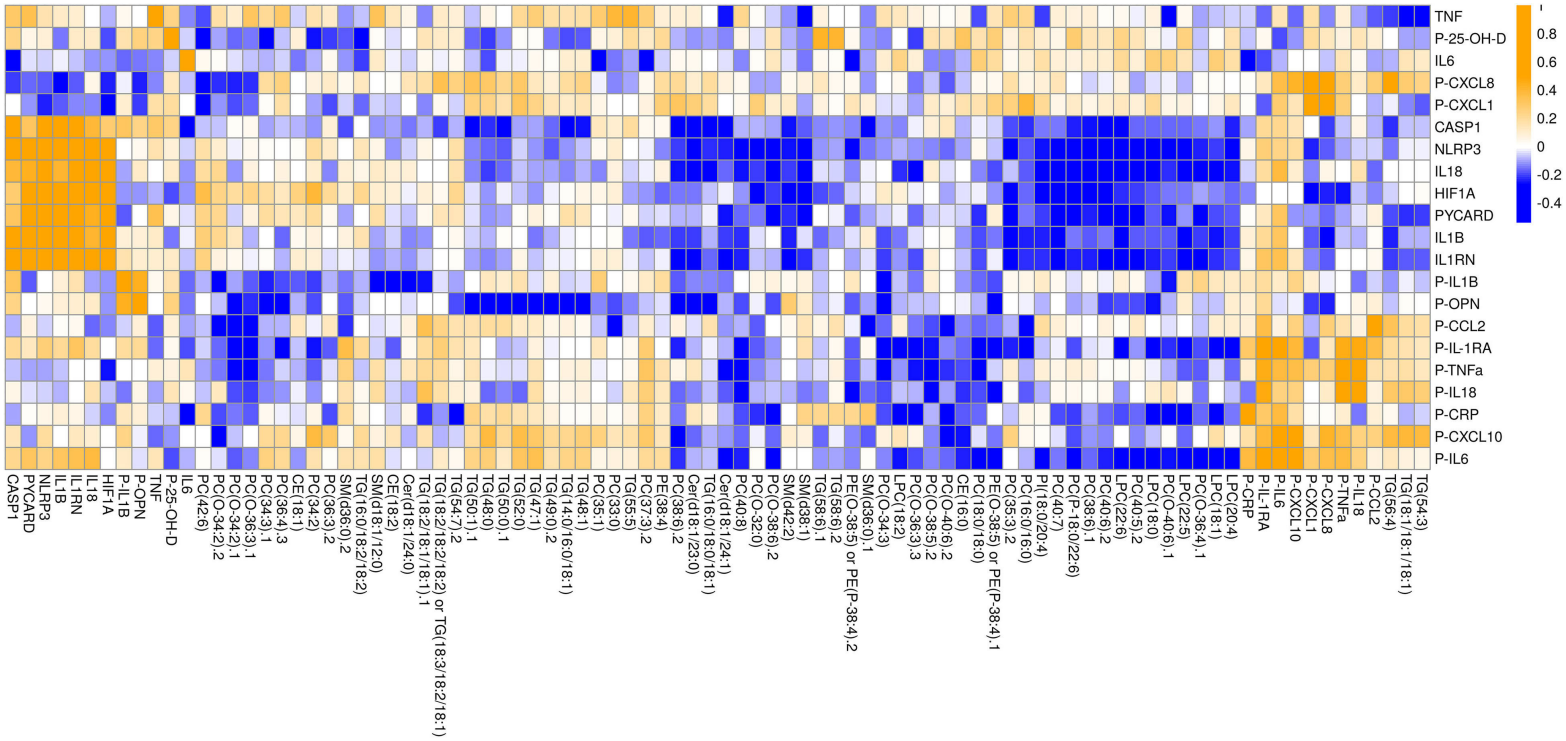
Inflammatory-mediator-centric heatmap of significant (*p* < 0.05 in ANOVA/TukeyHSD analyses) associations between inflammatory mediator and lipids, as measured by qPCR, electrochemiluminescent multiplex assay and UHPLC-QTOFMS. Lipid-lipid interactions were pruned from the original list of associations by assigning all lipid-lipid interactions with an NRR of 1. Circulating cytokines are indicated with P. Orange colour denote positive correlation and blue colour denotes negative correlations.

Following analysis of the plasma samples and data processing (see Methods), the lipidomics dataset included 185 molecular robustly-identified lipids. A total of 20 inflammatory markers were used in the analysis, of which 11 were measured as circulating cytokines and chemokines and 9 were analysed for their gene expression levels. The majority of these has been published in our earlier study (25). To elucidate disease-specific changes in lipids, the fold change (patient minus matched control) was calculated for downstream analysis. Figure 2 provides a network projection of the interactions between inflammatory factors and lipids. As shown in the network, the inflammasome-related gene expression variables (*NLRP3, PYCARD, CASP1, IL1RN, IL18, IL1B*) form an inflammasome-related core that are highly interconnected with positive correlations (R ≥ 0.5). This core also displays negative correlations to several lipids (*n* = 4) from different lipid classes (LPCs, PCs, Phosphatidylinositol (PI), Ceramides (Cer). Most inflammation-lipid interactions have negative correlations (73%), except for the chemokine CXCL10 whose interconnections are dominated by positive correlations (five positives and one negative). Another group of inflammation-lipid interactions was found with inflammation-related osteopontin (OPN), which exhibits negative associations with mainly triglycerides (seven negative correlations) (see Figures 2, 3).

### Identification of Lipid Candidates With Consistent Patterns Across Participants With Serious Mental Disorders

**Figure 4:**
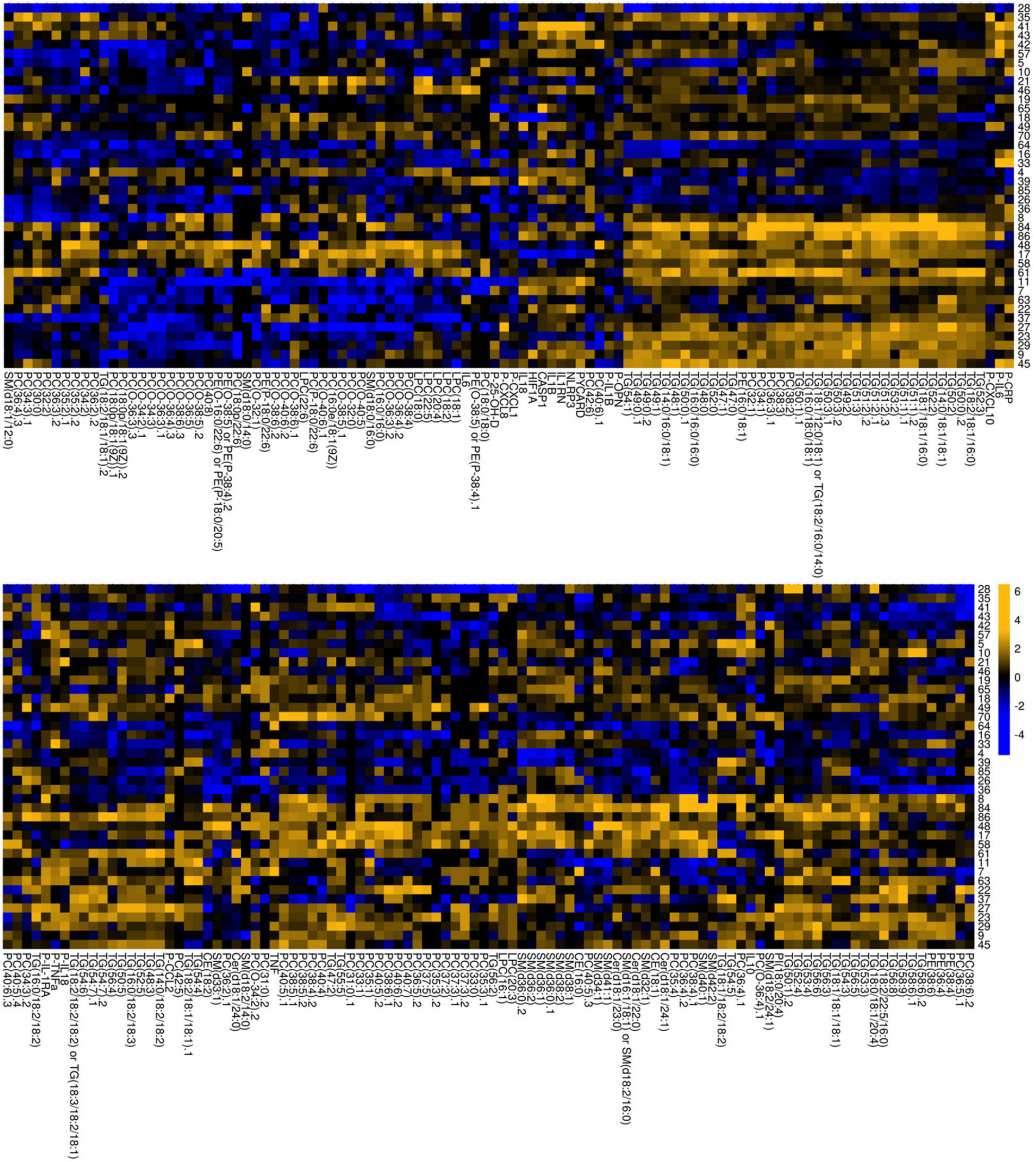
Heatmap of fold changes in lipid and inflammatory mediator levels. The fold-change of all lipids and inflammatory factors was calculated by subtraction of the log2, autoscaled values for all participants with mental disorder minus their respective, matched controls. Column legends indicate individual lipids and immune mediators [genes, circulating cytokines (indicated with P)]. Row legends indicate individual patient-control pairs.

**Figure 5:**
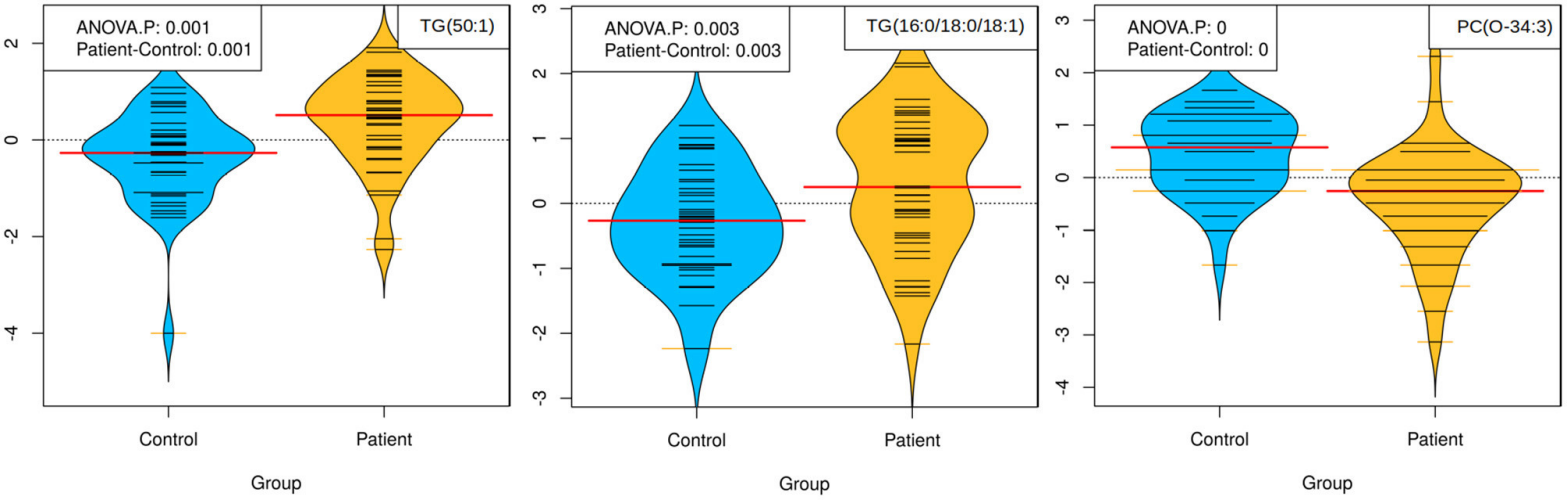
Beanplots showing the levels of TG (50:1), TG (16:0/18:0/18:1) and PC (O-34:3) in plasma collected from patients (*n* = 39) and controls (*n* = 39). The concentration of lipids followed was calculated based on lipid-class concentration curves. *p* < 0.05 was considered significant (ANOVA/TukeyHSD).

As shown in Figure 4, the participants with serious mental disorders displayed heterogeneous profiles as regard to lipid and inflammatory factor profiles. In light of this, further analyses were made to identify lipids that showed a consistent pattern of fold change within the cohort. This approach revealed a smaller set of strong (*R* > 0.5) correlations amongst inflammatory factors and lipids (Table 3). Among the seven identified lipid candidates, three of the lipids TG (50:1), TG (16:0/18:0/18:1) and PC (O-34:3) also showed significant differences between patients and controls, TG (50:1) and TG (16:0/18:0/18:1) were higher among participants with serious mental disorder whereas PC (O-34:3) was lower compared to the controls (Figure 5). Taken together, these three lipids show strong correlation with immune markers across all participants with serious mental disorder, as well as significantly differ compared to matched, healthy controls.

**Table 3:** Lipid candidates with strong inflammation-centric correlations across all patients.

**Feature 1**	**Feature 2**	**Correlation coefficient, R**
CXCL8 (Plasma)	TG (56:4)	0.523
OPN (Plasma)	TG (16:0/18:0/18:1)	−0.502
IL-6 (Plasma)	LPC (22:5)	−0.505
*CASP1* (Gene)	Cer (d18:1/23:0)	−0.505
IL-1Ra (Plasma)	PC (O-34:3)	−0.512
OPN (Plasma)	TG (50:1)	−0.512
IL-1Ra (Plasma)	PE (O-38:5) or PE (P-38:4)	−0.543

## Discussion

By systematically exploring the interactions between inflammatory mediators and lipids, we identified three strong immune-lipid interactions across the studied transdiagnostic cohort of participants with serious mental disorders. The correlation network (Figure 2) showed expected positive correlations between various inflammatory mediators, while almost three quarters of the immune-lipid interactions showed negative correlations. Among the inflammatory mediators that were most strongly associated with differences in lipid levels (Table 3), IL-6, IL-1Ra and *CASP1* have previously been found to be increased in the current cohort (26).

IL-1Ra, the receptor antagonist of IL-1, was negatively correlated with PC (O-34:3); the latter was moreover lower among participants with serious mental disorder compared to the controls. PC (O-34:3) is an ether phospholipid, i.e., consisting of an alkyl chain attached by an ether bond at the sn-1 position of the glycerol backbone (45, 46). The current study, with a decreased level of the ether phospholipid PC (O-34:3) in participants with serious mental disorder, is in line with previous research on SSD (47, 48). Ether phospholipids have an ability to act as endogenous antioxidants and thus counteract oxidative stress (45). Oxidative stress has been implicated in the pathology of several mental disorders such as SSD (49), ASD (50), and depression (51) and may cause a number of unwanted processes in the body, including oxidation of lipids. Oxidation of lipids, for example oxidation of PCs, can, in turn, induce inflammation through e.g., activation of the inflammasome, leading to release of the proinflammatory cytokines IL-1β and IL-18 (52). While in the present study, the inflammasome components formed a highly interconnected core with positive correlations, oxidative stress in the form of PC (O-34:3) decrease was only weakly (R <0.12) associated with central components in the inflammasome system. Instead, interactions between PC(O-34:3) and the IL-1 family of cytokines seem to be mediated *via* IL-1Ra. In addition, several other ether phospholipids showed correlations with IL1-Ra (Figure 2), but these changes had R values below 0.5. Elevated IL1-Ra is a persistent feature of individuals across multiple psychiatric diagnoses (53, 54). The current study suggests that this feature is closely related to changes in ether phospholipid levels, reflecting oxidative stress.

The two triacylglycerols, TG (16:0/18:0/18:1) and TG (50:1), were found to be increased in participants with serious mental disorder as compared to healthy controls. Increased levels of TGs in individuals with a first episode of psychosis have been reported previously (55, 56). In particular, increased levels of TGs of low carbon number and double bond count, such as TG (16:0/18:0/18:1), have been associated with non-alcoholic fatty liver disease (NAFLD) (57), and with weight gain and metabolic comorbidities in patients with first episode psychosis (58). In addition, Solberg *et al*. found higher levels of triglycerides in both the acute and the chronic stages of psychosis, as well as an association between higher levels in the acute stage and more severe symptoms in the chronic stage (59).

While TGs are not currently known to directly impact inflammatory processes, the two aforementioned TGs had a strong inverse association with OPN levels. OPN is mainly known as a proinflammatory mediator, previously linked to neurological disorders such as multiple sclerosis and Alzheimer's disease, where it is highly expressed in stressed microglia (60). Furthermore, elevated levels of OPN have been found in patients with SSD (61). Notably, OPN has also been found to have several protective and regenerative effects in tissues including the liver and CNS (62, 63). In this study OPN did not differ significantly between participants with serious mental disorders and controls (data not shown), indicating that the negative association between the identified triglycerides and OPN in individuals with serious mental disorders needs further investigation.

The current study has some important limitations, particularly small size of the study cohort. However, we made an effort to only include low-functioning individuals with serious mental disorders, representing clinical reality. We did not include moderately or mildly ill individuals or individuals with acceptable overall functioning. As with most transdiagnostic studies, a major limitation is the high level of heterogeneity, which is a reflection of the comorbidity. In fact, the high level of comorbidity was expected; previous studies have shown that 45 % of individuals with serious mental disorders fulfil the criteria for more than one diagnosis during 1 year (64) and individuals who develop chronic disorders with a severe course are particularly prone to fulfil a number of psychiatric diagnoses (4). For example, a child with OCD may be diagnosed with ASD in early teens (65) and then develop a psychosis in young adulthood, leading to a difficult to treat schizo-obsessive disorder (66). Self-harm, such as cutting oneself, is rather common among teenage girls, but in a small subset of susceptible individuals, usually with extensive comorbidity, it may become severe and require inpatient care, such as our participants with NSSID. Whereas, a focus on severity leads to comorbidity and heterogeneity, our included individuals suffered from one or several psychiatric disorders known to be associated with inflammation: SSD, ASD, OCD and depression. Nevertheless, the comorbidity and the heterogeneity make the results difficult to compare with studies that focus on one single diagnosis. Unfortunately, the small sample size of our study does not allow reliable subgroup analyses. Our approach enabled inclusion of very low functioning individuals with few treatment options and poor outcome. These individuals are unlikely to be accepted in treatment studies but represent the core of seriously ill in psychiatric care. Due to the heterogeneity and small sample size, care was taken to identify associations that were consistent across all participants and using statistical analyses that takes into account the sample size (e.g., TukeyHSD).

Most of our participants received medication. Out of the 39 participants, 18 were treated with antipsychotic medication, including participants with different primary diagnoses. Antipsychotics are commonly used, not only in SSD, but also in ASD, severe OCD and among individuals with self-harming behaviours (67–69). Antipsychotics, are known to affect lipids (56). however in the present study, no lipids showed a significant relationship with antipsychotic medication, including clozapine, (explored by using generalised linear models (GLM) on both pre- and post-processed data) and thus this potential confounder was dropped from the analysis. However, the inability to identify this confounder could be explained by the small sample size. Future research should ideally include much larger samples than ours.

Lifestyle factors such as diet and exercise are also likely to influence the lipids but were not accounted for in this study. Individuals suffering from serious mental disorders are known to be prone to unhealthy diet due to a number of reasons, i.e., impulsivity, craving of carbohydrates, side-effects of medications, and poverty. Unfortunately, these known differences in lifestyle patterns between groups are difficult to avoid. We did however carefully match participants with serious mental disorder and controls with respect to age and sex, and sampling was performed when participants were fasting, within the same hours of the day and with minimal handling times.

Taken together this exploratory study identified consistent disease-related alterations in the lipidome in a small cohort of low-functioning individuals with serious mental disorders who have previously been shown to exhibit increased levels of inflammatory markers. In particular, changes in two triglycerides and one ether phospholipid were strongly (*R* > 0.5) associated with OPN and IL-1Ra. While this remains to be studied in larger transdiagnostic cohorts, this study showed a mental illness-linked interplay between lipids and inflammatory factors and suggests that low-functioning individuals with mental disorders from different diagnostic groups, despite various observable differences, may also share a subset of common, transdiagnostic changes in immune-lipid pathways.

## Data Availability Statement

The raw data supporting the conclusions of this article will be made available by the authors, without undue reservation.

## Ethics Statement

The studies involving human participants were reviewed and approved by Regional Ethical Review Board in Uppsala, Sweden (2016/091). Written informed consent to participate in this study was provided by the participant's legal guardian/next of kin.

## Author Contributions

ES, SB, and MH: conceptualisation. DE, AM, UH, and TH: investigation. DE, MO, AM, UH, and TH: methodology. AM and TH: formal analysis. AM: visualisation. DE, AM, and UH: writing-original draft. DE, MO, AM, UH, SB, MH, TH, and ES: data discussion and writing–review and editing. DE, MO, SB, and ES: supervision. All authors approved the final manuscript.

## Funding

The work was supported by research grants awarded from Region Örebro County (ALF-funding OLL-930309 and from the Research Committee, OLL-834251). The funding sources had no role in study design, data collection, analyses or interpretation of the data.

## Conflict of Interest

The authors declare that the research was conducted in the absence of any commercial or financial relationships that could be construed as a potential conflict of interest.

## Publisher's Note

All claims expressed in this article are solely those of the authors and do not necessarily represent those of their affiliated organizations, or those of the publisher, the editors and the reviewers. Any product that may be evaluated in this article, or claim that may be made by its manufacturer, is not guaranteed or endorsed by the publisher.

## References

[1] Vigo D. Thornicroft G, Atun R. Estimating the true global burden of mental illness. Lancet Psychiatry. (2016) 3:171–8. 10.1016/S2215-0366(15)00505-2 26851330

[2] Plana-Ripoll O Pedersen CB Agerbo E Holtz Y Erlangsen A Canudas-Romo V. A comprehensive analysis of mortality-related health metrics associated with mental disorders: a nationwide, register-based cohort study. Lancet. (2019) 394:1827–35. 10.1016/S0140-6736(19)32316-5 31668728

[3] Association AP. Diagnostic and Statistical Manual of Mental Disorders, 5th Ed. (DSM-5).Washington, DC: American Psychiatric Association (2013).

[4] Plana-Ripoll O Pedersen CB Holtz Y Benros ME Dalsgaard S de Jonge P. Exploring comorbidity within mental disorders among a Danish national population. JAMA Psychiatry. (2019) 76:259–70. 10.1001/jamapsychiatry.2018.3658 30649197 PMC6439836

[5] Maj M. Why the clinical utility of diagnostic categories in psychiatry is intrinsically limited and how we can use new approaches to complement them. World Psychiatry. (2018) 17:121–2. 10.1002/wps.20512 29856539 PMC5980436

[6] Mansell W. Transdiagnostic psychiatry goes above and beyond classification. World Psychiatry. (2019) 18:360–1. 10.1002/wps.20680 31496093 PMC6732685

[7] Miller AH Raison CL. The role of inflammation in depression: from evolutionary imperative to modern treatment target. Nat Rev Immunol. (2016) 16:22–34. 10.1038/nri.2015.5 26711676 PMC5542678

[8] Miller BJ Goldsmith DR. Towards an immunophenotype of schizophrenia: progress, potential mechanisms, and future directions. Neuropsychopharmacology. (2017) 42:299–317. 10.1038/npp.2016.211 27654215 PMC5143505

[9] Meltzer A Van de Water J. The role of the immune system in autism spectrum disorder. Neuropsychopharmacology. (2017) 42:284–98. 10.1038/npp.2016.158 27534269 PMC5143489

[10] Attwells S Setiawan E Wilson AA Rusjan PM Mizrahi R Miler L. Inflammation in the neurocircuitry of obsessive-compulsive disorder. JAMA Psychiatry. (2017) 74:833–40. 10.1001/jamapsychiatry.2017.1567 28636705 PMC5710556

[11] Yuan N Chen Y Xia Y Dai J Liu C. Inflammation-related biomarkers in major psychiatric disorders: a cross-disorder assessment of reproducibility and specificity in 43 meta-analyses. Transl Psychiatry. (2019) 9:233. 10.1038/s41398-019-0570-y 31534116 PMC6751188

[12] Rodrigues-Amorim D Rivera-Baltanas T Spuch C Caruncho HJ Gonzalez-Fernandez A Olivares JM. Cytokines dysregulation in schizophrenia: a systematic review of psychoneuroimmune relationship. Schizophr Res. (2017) 197:19–33. 10.1016/j.schres.2017.11.023 29239785

[13] Masi A Quintana DS Glozier N Lloyd AR Hickie IB Guastella AJ. Cytokine aberrations in autism spectrum disorder: a systematic review and meta-analysis. Mol Psychiatry. (2015) 20:440–6. 10.1038/mp.2014.59 24934179

[14] Mondelli V Vernon AC Turkheimer F Dazzan P Pariante CM. Brain microglia in psychiatric disorders. Lancet Psychiatry. (2017) 4:563–72. 10.1016/S2215-0366(17)30101-3 28454915

[15] Hussain G Anwar H Rasul A Imran A Qasim M Zafar S. Lipids as biomarkers of brain disorders. Crit Rev Food Sci Nutr. (2020) 60:351–74. 10.1080/10408398.2018.1529653 30614244

[16] Walther A Cannistraci CV Simons K Durán C Gerl MJ Wehrli S. Lipidomics in major depressive disorder. Front Psychiatry. (2018) 9:459. 10.3389/fpsyt.2018.00459 30374314 PMC6196281

[17] Castillo RI Rojo LE Henriquez-Henriquez M Silva H Maturana A Villar MJ. From molecules to the clinic: linking schizophrenia and metabolic syndrome through sphingolipids metabolism. Front Neurosci. (2016) 10:488. 10.3389/fnins.2016.00488 27877101 PMC5100552

[18] Orešič M Tang J Seppänen-Laakso T Mattila I Saarni SE Saarni SI. Metabolome in schizophrenia and other psychotic disorders: a general population-based study. Genome Med. (2011) 3:19. 10.1186/gm233 21429189 PMC3092104

[19] Wang D Cheng SL Fei Q Gu H Raftery D Cao B. Metabolic profiling identifies phospholipids as potential serum biomarkers for schizophrenia. Psychiatry Res. (2019) 272:18–29. 10.1016/j.psychres.2018.12.008 30579177

[20] Anand PK. Lipids, inflammasomes, metabolism, and disease. Immunol Rev. (2020) 297:108–22. 10.1111/imr.12891 32562313

[21] O'Neill LA Kishton RJ Rathmell J. A guide to immunometabolism for immunologists. Nat Rev Immunol. (2016) 16:553–65. 10.1038/nri.2016.70 27396447 PMC5001910

[22] Swanson KV Deng M Ting JP-Y. The NLRP3 inflammasome: molecular activation and regulation to therapeutics. Nat Rev Immunol. (2019) 19:477–89. 10.1038/s41577-019-0165-0 31036962 PMC7807242

[23] Meyers AK Zhu X. The NLRP3 Inflammasome: metabolic regulation and contribution to inflammaging. Cells. (2020) 9:1808. 10.3390/cells9081808 32751530 PMC7463618

[24] Snodgrass RG Huang S Choi IW Rutledge JC Hwang DH. Inflammasome-mediated secretion of IL-1β in human monocytes through TLR2 activation; modulation by dietary fatty acids. J Immunol. (2013) 191:4337–47. 10.4049/jimmunol.1300298 24043885 PMC3825708

[25] Karasawa T Kawashima A Usui-Kawanishi F Watanabe S Kimura H Kamata R. Saturated fatty acids undergo intracellular crystallization and activate the NLRP3 inflammasome in macrophages. Arterioscler Thromb Vasc Biol. (2018) 38:744–56. 10.1161/ATVBAHA.117.310581 29437575

[26] Hylén U Eklund D Humble M Bartoszek J Särndahl E Bejerot S. Increased inflammasome activity in markedly ill psychiatric patients: an explorative study. J Neuroimmunol. (2020) 339:577119. 10.1016/j.jneuroim.2019.577119 31786499

[27] Hellberg S Eklund D Gawel DR Köpsén M Zhang H Nestor CE. Dynamic response genes in CD4+ T cells reveal a network of interactive proteins that classifies disease activity in multiple sclerosis. Cell Rep. (2016) 16:2928–39. 10.1016/j.celrep.2016.08.036 27626663

[28] Sheehan DV Lecrubier Y Sheehan KH Amorim P Janavs J Weiller E. The mini-international neuropsychiatric interview (MINI): the development and validation of a structured diagnostic psychiatric interview for DSM-IV and ICD-10. J Clin Psychiatry. (1998) 59:22–33. 9881538

[29] Gratz KL Dixon-Gordon KL Chapman AL Tull MT. Diagnosis and characterization of DSM-5 nonsuicidal self-injury disorder using the clinician-administered nonsuicidal self-injury disorder index. Assessment. (2015) 22:527–39. 10.1177/1073191114565878 25604630 PMC5505727

[30] Guy W. ECDEU Assessment Manual for Psychopharmacology. Rockville, MD: USA US Department of Heath, Education, and Welfare Public Health Service Alcohol, Drug Abuse, and Mental Health Administration (1976).

[31] Jones SH Thornicroft G Coffey M Dunn G. A brief mental health outcome scale-reliability and validity of the Global Assessment of Functioning (GAF). Br J Psychiatry. (1995) 166:654–9. 10.1192/bjp.166.5.654 7620753

[32] Ustun TB Chatterji S Kostanjsek N Rehm J Kennedy C Epping-Jordan J. Developing the world health organization disability assessment schedule 2.0. Bull World Health Organ. (2010) 88:815–23. 10.2471/BLT.09.067231 21076562 PMC2971503

[33] Kroenke K Spitzer RL Williams JB. The PHQ-9: validity of a brief depression severity measure. J Gen Intern Med. (2001) 16:606–13. 10.1046/j.1525-1497.2001.016009606.x 11556941 PMC1495268

[34] Quilty LC Ayearst L Chmielewski M Pollock BG Bagby RM. The psychometric properties of the personality inventory for DSM-5 in an APA DSM-5 field trial sample. Assessment. (2013) 20:362–9. 10.1177/1073191113486183 23588687

[35] GBD 2016 Disease and Injury Incidence and Prevalence Collaborators. Global, regional, and national incidence, prevalence, and years lived with disability for 328 diseases and injuries for 195 countries, 1990-2016: a systematic analysis for the global burden of disease study 2016. Lancet. (2017) 390:1211–59. 10.1016/S0140-6736(17)32154-2 28919117 PMC5605509

[36] Tek C Ulug B Rezaki BG Tanriverdi N Mercan S Demir B. Yale-brown obsessive compulsive scale and us national institute of mental health global obsessive compulsive scale in Turkish: reliability and validity. Acta Psychiatr Scand. (1995) 91:410–3. 10.1111/j.1600-0447.1995.tb09801.x 7676839

[37] Latimer S Meade T Tennant A. Measuring engagement in deliberate self-harm behaviours: psychometric evaluation of six scales. BMC Psychiatry. (2013) 13:4. 10.1186/1471-244X-13-4 23286337 PMC3605243

[38] Folch J Lees M Sloane Stanley GH. A simple method for the isolation and purification of total lipides from animal tissues. J Biol Chem. (1957) 226:497–509. 10.1016/S0021-9258(18)64849-5 13428781

[39] McGlinchey A Sinioja T Lamichhane S Bodin J Siljander H Geng D. Prenatal Exposure To Environmental Chemicals Modulates Serum Phospholipids In Newborn Infants, Increasing Later Risk Of Type 1 Diabetes. bioRxiv. (2019).

[40] R: A Language and Environment for Statistical Computing (2017).

[41] Wei T Simko VR. Package “corrplot”: Visualization of a Correlation Matrix. Version 089 ed (2017).

[42] Roverato A Castelo R. The networked partial correlation and its application to the analysis of genetic interactions. J R Stat Soc Ser C Appl Stat. (2017) 66:647–65. 10.1111/rssc.12166

[43] Hansen K Gentry J Long L Gentleman R Falcon S Hahne F. Rgraphviz: Provides Plotting Capabilities For R Graph Objects. R package version 2360 ed. (2021).

[44] Kampstra P. Beanplot: a boxplot alternative for visual comparison of distributions. J Stat Softw. (2008) 28:1–9. 10.18637/jss.v028.c0127774042

[45] Dean JM Lodhi IJ. Structural and functional roles of ether lipids. Protein Cell. (2018) 9:196–206. 10.1007/s13238-017-0423-5 28523433 PMC5818364

[46] Dorninger F Forss-Petter S Berger J. From peroxisomal disorders to common neurodegenerative diseases-the role of ether phospholipids in the nervous system. FEBS Lett. (2017) 591:2761–88. 10.1002/1873-3468.12788 28796901 PMC5856336

[47] Tessier C Sweers K Frajerman A Bergaoui H Ferreri F Delva C. Membrane lipidomics in schizophrenia patients: a correlational study with clinical and cognitive manifestations. Transl Psychiatry. (2016) 6:e906. 10.1038/tp.2016.142 27701405 PMC5315538

[48] Wood PL Unfried G Whitehead W Phillipps A Wood JA. Dysfunctional plasmalogen dynamics in the plasma and platelets of patients with schizophrenia. Schizophr Res. (2015) 161:506–10. 10.1016/j.schres.2014.11.032 25497441

[49] Fraguas D Díaz-Caneja CM Ayora M Hernández-Álvarez F Rodríguez-Quiroga A Recio S. Oxidative stress and inflammation in first-episode psychosis: a systematic review and meta-analysis. Schizophr Bull. (2019) 45:742–51. 10.1093/schbul/sby125 30169868 PMC6581144

[50] Bjørklund G Meguid NA El-Bana MA Tinkov AA Saad K Dadar M. Oxidative stress in autism spectrum disorder. Mol Neurobiol. (2020) 57:2314–32. 10.1007/s12035-019-01742-2 32026227

[51] Yager S Forlenza MJ Miller GE. Depression and oxidative damage to lipids. Psychoneuroendocrinology. (2010) 35:1356–62. 10.1016/j.psyneuen.2010.03.010 20417039

[52] Yeon SH Yang G Lee HE Lee JY. Oxidized phosphatidylcholine induces the activation of NLRP3 inflammasome in macrophages. J Leukoc Biol. (2017) 101:205–15. 10.1189/jlb.3VMA1215-579RR 27256568

[53] Goldsmith DR Rapaport MH Miller BJ. A meta-analysis of blood cytokine network alterations in psychiatric patients: comparisons between schizophrenia, bipolar disorder and depression. Mol Psychiatry. (2016) 21:1696–709. 10.1038/mp.2016.3 26903267 PMC6056174

[54] Mørch RH Dieset I Faerden A Hope S Aas M Nerhus M. Persistent increase in TNF and IL-1 markers in severe mental disorders suggests trait-related inflammation: a one year follow-up study. Acta Psychiatr Scand. (2017) 136:400–8. 10.1111/acps.12783 28815548

[55] Misiak B Stańczykiewicz B Łaczmański Ł Frydecka D. Lipid profile disturbances in antipsychotic-naive patients with first-episode non-affective psychosis: a systematic review and meta-analysis. Schizophr Res. (2017) 190:18–27. 10.1016/j.schres.2017.03.031 28325572

[56] Pillinger T Beck K Stubbs B Howes OD. Cholesterol and triglyceride levels in first-episode psychosis: systematic review and meta-analysis. Br J Psychiatry. (2017) 211:339–49. 10.1192/bjp.bp.117.200907 28982658 PMC5709673

[57] Orešič M Hyötyläinen T Kotronen A Gopalacharyulu P Nygren H Arola J. Prediction of non-alcoholic fatty-liver disease and liver fat content by serum molecular lipids. Diabetologia. (2013) 56:2266–74. 10.1007/s00125-013-2981-2 23824212 PMC3764317

[58] Suvitaival T Mantere O Kieseppä T Mattila I Pöhö P Hyötyläinen T. Serum metabolite profile associates with the development of metabolic co-morbidities in first-episode psychosis. Transl Psychiatry. (2016) 6:e951. 10.1038/tp.2016.222 27845774 PMC5314133

[59] Solberg DK Bentsen H Refsum H Andreassen OA. Association between serum lipids and membrane fatty acids and clinical characteristics in patients with schizophrenia. Acta Psychiatr Scand. (2015) 132:293–300. 10.1111/acps.12388 25597473

[60] Yu H Liu X Zhong Y. The effect of osteopontin on microglia. Biomed Res Int. (2017) 2017:1879437. 10.1155/2017/1879437 28698867 PMC5494082

[61] Kovács M Tényi T Kugyelka R Prenek L Hau L Magyar ÉE. Elevated osteopontin and interferon gamma serum levels and increased neutrophil-to-lymphocyte ratio are associated with the severity of symptoms in schizophrenia. Front Psychiatry. (2019) 10:996. 10.3389/fpsyt.2019.00996 32038330 PMC6989480

[62] Patouraux S Rousseau D Rubio A Bonnafous S Lavallard VJ Lauron J. Osteopontin deficiency aggravates hepatic injury induced by ischemia-reperfusion in mice. Cell Death Dis. (2014) 5:e1208. 10.1038/cddis.2014.174 24810044 PMC4047890

[63] Cappellano G Vecchio D Magistrelli L Clemente N Raineri D Barbero Mazzucca C. The Yin-Yang of osteopontin in nervous system diseases: damage versus repair. Neural Regen Res. (2021) 16:1131–7. 10.4103/1673-5374.300328 33269761 PMC8224140

[64] van Loo HM Romeijn JW. Psychiatric comorbidity: fact or artifact? Theor Med Bioeth. (2015) 36:41–60. 10.1007/s11017-015-9321-0 25636962 PMC4320768

[65] Martin AF Jassi A Cullen AE Broadbent M Downs J Krebs G. Co-occurring obsessive-compulsive disorder and autism spectrum disorder in young people: prevalence, clinical characteristics and outcomes. Eur Child Adolesc Psychiatry. (2020) 29:1603–11. 10.1007/s00787-020-01478-8 32008168 PMC7595977

[66] Tezenas du Montcel C Pelissolo A Schürhoff F Pignon B. Obsessive-compulsive symptoms in schizophrenia: an up-to-date review of literature. Curr Psychiatry Rep. (2019) 21:64. 10.1007/s11920-019-1051-y 31263973

[67] Jobski K Höfer J Hoffmann F Bachmann C. Use of psychotropic drugs in patients with autism spectrum disorders: a systematic review. Acta Psychiatr Scand. (2017) 135:8–28. 10.1111/acps.12644 27624381

[68] Veale D Miles S Smallcombe N Ghezai H Goldacre B Hodsoll J. A typical antipsychotic augmentation in SSRI treatment refractory obsessive-compulsive disorder: a systematic review and meta-analysis. BMC Psychiatry. (2014) 14:317. 10.1186/s12888-014-0317-5 25432131 PMC4262998

[69] Turner BJ Austin SB Chapman AL. Treating nonsuicidal self-injury: a systematic review of psychological and pharmacological interventions. Can J Psychiatry. (2014) 59:576–85. 10.1177/070674371405901103 25565473 PMC4244876

